# Ethical Challenges Related to the Novel Coronavirus (COVID-19) Outbreak: Interviews With Professionals From Saudi Arabia

**DOI:** 10.3389/fmed.2021.620444

**Published:** 2021-03-30

**Authors:** Ghiath Alahmad, Hanie Richi, Ala'a BaniMustafa, Adel F. Almutairi

**Affiliations:** King Abdullah International Medical Research Center, King Saud bin Abdulaziz University for Health Sciences, Ministry of National Guard Health Affairs, Riyadh, Saudi Arabia

**Keywords:** ethical challenges, COVID-19, professionals, ethical principles, Saudi Arabia

## Abstract

The new and dangerous coronavirus disease (COVID-19) has posed a serious challenge to the ability of healthcare systems of many countries to contain the spread of the disease and to mitigate its various consequences. The disease posed many ethical challenges both in itself and in the methods used in its management. Although the ethical principles that healthcare operates under are universal, a thorough understanding of the ethical difficulties it poses necessitates consideration of contextual, societal, and cultural factors. This study provides an in-depth exploration of the ethical challenges related to the COVID-19 pandemic outbreak in relation to healthcare providers, medical researchers, and decision-makers in Saudi Arabia. Four themes were extracted from participants' responses, namely, ethical challenges about disease-control measures, challenges to actions in certain groups, challenges regarding software programs, and finally ethics in research practices. Each theme likewise contained sub-themes. The themes and sub-themes were discussed in light of the ethical principles: autonomy, beneficence, non-beneficence, and justice, as well as other principles, such as protecting confidentiality, privacy, and preventing stigma and discrimination.

## Background

Novel diseases, such as Middle East Respiratory Syndrome and Severe Acute Respiratory Syndrome, that appeared within the past 20 years received interest in societies all around the globe. The new and dangerous disease COVID-19, which had its first outbreak bin December 2019 in Wuhan, China, and is known to cause severe pneumonia, has shaken healthcare systems all over the world (1). This alarmingly contagious virus is known to spread from one person to another through direct close contact in a similar manner to influenza, through respiratory droplets released when the infected person coughs or sneezes (2). Recently, coronavirus has spread to many parts of the world, through the travelers and Affected more than 41,093,074 people in 217 countries and the countries in the Gulf Cooperation Council (GCC) have 897,790 cases (as of October 21, 2020) (3–5). The death toll has reached 4,702 people, and unfortunately this figure is increasing. The World Health Organization officially assigned the outbreak to pandemic status on March 11, 2020.

The outbreak of COVID-19 has posed challenges to many countries and their ability to contain the spread of the virus. The evaluation of past experience with epidemic diseases can help shape the response to future challenges. Planning strategies to tackle infectious disease outbreaks and advance preparation can lead to a rapid and strategic response to create a road to recovery. However, logistical challenges are linked to the recognition and identification of infectious disease outbreaks, which can impede the planning of effective strategies for control (6). Among the ethical concerns, for example, is the outbreak of an unknown organism.

When the strain of a virus that is causing a certain infectious disease is unknown, it is impossible to know what vaccines or medications are most appropriate should be looked to for the development of a treatment plan to combat the outbreak. The first development plan for pandemic vaccine preparation and stocking prior to an infectious disease outbreak poses the challenge of what to research, develop, and keep and stock for a what-if situation, upon which it can be made available for distribution and administration before the strain becomes known. One example of this can be seen in the case of HIV in the 1980s, which could not be diagnosed at that time, due to the poor state of knowledge of the disease and its agent. It was not until the year 2003 that a better understanding of the nature of the virus became available (7). It was only at this point that drug development, effective testing, and more scientific diagnoses emerged. Recent years have shown advances in the testing of HIV vaccines in clinical trials (8). This is similar to the case of the novel coronavirus (COVID-19), in particular regarding to unavailability of a vaccine, which may raise ethical concerns among researchers.

Due to the unavailability of a COVID-19 vaccine and to time pressure, experiments may be conducted that do not follow approved and well-established procedures, such as the performance of a thorough series of animal experiments before testing on humans. Even after an effective vaccine is developed, healthcare facilities will doubtless face a shortage vaccine due to the need for them all over the world. This poses the following question: how should healthcare organizations and their regulators handle this crisis? A rubric according to which the limited supply of vaccines can be administered to those who matter most is necessary. A potential order of prioritization might run as follows:

Healthcare practitioners who in direct contact with patients should be the first priority, followed by citizens at the highest risk, such as 65-year-olds who are already ill, and then individuals who have been hospitalized more broadly (8).

It is likely that in the case of an infectious disease outbreak, such as that of COVID-19, it can overwhelm medical care systems, as was seen in Italy, for example. In particular, the number of patients who needed ventilation outstripped intensive care unit capacity in many parts of Italy (9). This prompted criticism of government planners along both ethical and legal dimensions in regard to their failure to predict and adapt to the possibility of a pandemic disease (10). The most pressing matter here is deciding what to do when it reaches the point where hospitals cannot accept patients due to their capacities having been overwhelmed.

One potential remedy is to make alternative sites available and to use equipped volunteer caregivers. In addition, agencies should be most concerned about patients who have limited or even no health insurance coverage; that is, they should must respond with kindness, not simply leave patients to die. The challenge here is to honor the commitment to patient well-being and consequently, to the patient's civil rights.

Epidemic and pandemic disease outbreaks inevitably impact a country's economy negatively. It is necessary for public health planning and preparedness take into account death and citizens in a state of despair (7, 11–15). A major role that they play is taking cost into consideration and assigning the balance between benefit and risk. This entails the provision of appropriate healthcare needs that include agreement on fair procedures and conformity to legal rights, while ensuring a reasonable budget to keep disease under control.

Screening procedures to support early diagnosis and treatment are critical in an epidemic outbreak to patient recovery and to prevent further disease spread and to moderate government expenditure (6).

Most people who think that they have had exposure will voluntarily agree to be tested, but some may pose difficulties on this point, presenting a challenge for Public Health Services or healthcare authorities, who may have to authorize mandatory testing. This authorization, however, should only be used if voluntary and advisory means are unsuccessful.

In response to the effort to contain the spread of the debilitating infectious disease outbreak of COVID-19 and other potential pandemics or outbreaks that may follow, healthcare authorities and policy makers in many countries issued a statement that affected individuals should be quarantined or subjected to involuntary confinement (15). The ethical challenge here is in justifying how harmful it would be for an affected individual to mingle with the public at large. Quarantine may be appropriate if the disease is highly contagious. At this moment, it is mandatory that the individual to be quarantined has access to necessities, such as medical care and food and to be informed about his/her family to maintain quality of life. Here, it is important for there to be a balance between the spread of a disease and freedom from restrictions.

The question of whether it is appropriate to name quarantined individuals in the media is also concerning. From a public health practice point of view, this should be done because it allows doctors to trace infected individuals' location, perform medical evaluation if necessary, and help them with their treatment if needed. Individuals, however, must be granted the right to maintain their privacy (15, 16).

During the present COVID-19 pandemic, one way to prevent disease spread is to cancel public events, closing schools, workplaces, sporting venues, and restaurants (17). Doing this can be quite challenging, as school closings and rescheduling make it difficult to cover planned lessons. Further, such closures can produce immense economic impacts, especially on businesses such as restaurants, shopping centers, and entertainment venues.

Saudi Arabia has is observing the pandemic with great interest and conducting important research on COVID-19, and Saudi universities and research centers are establishing research programs and redirecting the financial support necessary to such efforts. Saudi Arabia ranked twenty-fifth in the world and first in the Arab world in terms of its scientific publishing on COVID-19 (18), searching PubMed (on October 20, 2020) showed that there are 677 scientific papers published by researchers in Saudi Arabia, although none of these covered the ethical aspects of the pandemic. Saudi officials did attempt to establish ethical controls in the response to and management of COVID-19, as well as in researching it, particularly with regard to the National Committee of Bioethics, which issued a statement on research controls in times of epidemics and emergencies.

In this research project, we investigate ethical issues related to the COVID-19 outbreak. These findings will help address ethical questions at the individual, social, and organizational levels and will help develop guidelines that can be used by fellow clinicians, researchers, and policy makers in Saudi Arabia. We especially focus on the following issues, which are important but little researched issues in the Middle East.

## Methods

### Study Design

This study adopted a qualitative research approach. In order to explore ethical challenges related to the COVID-19 pandemic in depth among professionals in Saudi Arabia, we conducted 24 interviews, with frontline healthcare providers (physicians and nurses) who specialize in treating infectious diseases, in addition to stakeholders and experts in the programs that manage COVID-19 outbreak, such as researchers and decision makers in Saudi Arabia, in King Abdul-Aziz Medical City, which includes King Abdullah Specialized Hospital and King Abdullah International Medical Research Center, in Riyadh, Saudi Arabia. Purposive sampling was used to recruit the participants (19), face to face interviews were conducted until saturation was reached (20). The interviews were conducted between May and September 2020. In order to identify the themes of importance, three interviews with three professional from different backgrounds were conducted. None of these three interviews were included in this study.

### Participants and Data Collection

A phenomenological approach was used to collect data (19). Each semi-structured interview was conducted over 45–60 min. We began each encounter by giving a description of this study, explaining its objectives, assuring participants of their confidentiality, and asking for voluntary informed consent. The interviews used open-ended questions, following the interview guide, and began with demographic data. The interview questions were used to cover ethical considerations in relation to these topics, among others: outbreak of an unknown disease agent, vaccine shortages, drug treatment, experimental drugs, care shortages, occasions for screening, quarantine for suspected or confirmed infection, confidentiality and privacy, airlifts, travel restrictions, and cancelation of public events.

The interviews were conducted face to face and were audio-taped and transcribed. The interviews were conducted in places convenient to the interviewees.

### Data Analysis

The inductive approach to qualitative data analysis was employed, which revolves around finding patterned meaning in data (21). Several particular steps were used, which including coding, searching, reviewing, defining themes, and identifying them. NVivo 11 software was used for analysis. The analysis was performed iteratively, which required the analyzer to move back and forth across the listed actions. The collected data were analyzed separately by the research team, and then the outcomes were compared to strengthen the analysis. Four main themes were produced by the analyses, and each theme contained subthemes.

### Ethical Considerations and Consent Documents

Ethical approval was obtained from the IRB office at King Abdullah International Medical Research Centre KAIMRC, and informed consents were collected before starting the interviews. No identifier will be used, and privacy and confidentiality will be completely protected. Appropriate informed consent for qualitative research will be used.

## Results

### Participant Characteristics

Individual Interviews were conducted with 24 healthcare providers and researchers working in Riyadh, Saudi Arabia, at King Abdullah International Medical Research Center, King Saud bin Abdulaziz University for Health Sciences, and King Abdullah Specialized Hospital in the National Guard for Health Affairs. All of the interviewees expressed their interest in working on COVID-19, describing it, managing it, and conducting medical research on it.

Nine of the participants were specialists in infectious diseases, five worked in experimental medicine, and two worked in intensive care units. Others had positions at laboratories where samples from COVID-19 patients were tested. Seven participants were IRB members who had examined research proposals for studies of COVID-19.

Seven of the participants were female, and the remainder were male. The interviews were with a variety of ages ranging from 30 to 60 years. Finally, 14 were Saudis, and the others were non-Saudis of different nationalities (Table 1).

**Table 1:** Characteristics of participants.

**#**	**Sex**	**Nationality**	**Job Title**	**Specialty**	**Institute**	**Department**
1	M	Non-Saudi	Research Scientist	Infectious Disease	KAIMRC	Medical Genomics Research
2	F	Saudi	Post-Doctoral Researcher	Infectious Disease	KAIMRC	Infectious Disease
3	F	Non-Saudi	Research Associate	Experimental Medicine	KAIMRC	Experimental Medicine
4	M	Saudi	Adjunct Research Scientist, Infectious Diseases Research	Infectious Disease	KAIMRC	Infectious Disease
5	M	Saudi	Post-Doctoral Researcher	Experimental Medicine	KAIMRC	Experimental Medicine
6	M	Saudi	Operations Administrator Laboratory Services	Laboratory	KAIMRC	Research Operation
7	M	Non-Saudi	Senior Research Scientist	Infectious Disease	KAIMRC	Nanomedicine
8	M	Non-Saudi	Associate Research Scientist	Experimental Medicine	KAIMRC	Experimental Medicine
9	M	Non-Saudi	Chairman, Senior Research Scientist	Infectious Disease	KAIMRC	Medical Research Core Facility
10	M	Non-Saudi	Research Scientist	Experimental Medicine	KAIMRC	Experimental Medicine
11	M	Non-Saudi	Clinical Research Coordinator	Infectious Disease	KAIMRC	Research Office
12	M	Non-Saudi	Senior Research Scientist	Experimental Medicine	KAMC	Experimental Medicine
13	M	Non-Saudi	Clinical Research Coordinator	IRB	KAIMRC	Institutional Review Board
14	M	Saudi	ICU consultant	Intensive Care Unit	KAMC	Intensive Care Unit
15	M	Saudi	ID Consultant	Infectious Disease	KAMC	Infectious Disease
16	M	Saudi	Professor of Genetics	Infectious Disease	KFSH	KFSH & RC
17	M	Non-Saudi	ICU consultant	Intensive Care Unit		Intensive Care Unit
18	F	Saudi	Associate Professor	IRB/Pharmacology	KSAU-HS	College of Pharmacy,KSAU-HS
19	M	Saudi	Chairman, Infectious Disease Research	Infectious Disease	KAIMRC	Infectious Disease Research Unit
20	M	Saudi	Assistant Professor Physiology	IRB/Physiology	SSAU	College of Medicine,KSAU-HS
21	F	Saudi	Assistant Professor of Pharmacology and Toxicology	IRB/Pharmacology	KSAU-HS	College of Pharmacy, KSAU-HS
22	F	Saudi	Consultant	IRB/Dental	KAMC	Orthodontics Division, Dental Service
23	F	Saudi	Consultant	IRB/Gynecology	KASCH	Division of Gynecology Oncology, Department of Oncology
24	F	Saudi	Consultant	IRB/Pediatric	KASCH	Pediatric, Intensivist–PICU-KASCH

### Themes

Four main themes were found, each with a number of subthemes (Table 2).

**Table 2:** Themes and sub-themes.

Theme 1: Ethical challenges regarding measures taken to control COVID-19	Preventing public gatherings and applying restrictions
	Closing schools and change teaching methods
	Work changes in institutions and companies
	Banning Communal Prayer, the Umrah, and the Hajj
Theme 2: Ethical challenges regarding procedures and actions for certain groups	Screening of certain groups
	Isolation
	Protecting volunteers and healthcare providers
Theme 3: Ethical challenges of detecting COVID-19 and confidentiality issues	Software programs
	Exposing infected people names
Theme 4: Ethical challenges in COVID-19 research	Quantity of research and publication
	Uncertainty
	Resource distribution
	Not following correct experimental path

#### Theme 1: Ethical Challenges Regarding Measures Taken to Control COVID-19

##### Preventing public gatherings and applying restrictions

The majority of participants were positive about the actions and decisions that the government took to ban various types of gathering. One participant explained:

“The measures taken by government to close down many economic activities and prevent social gatherings were necessary, and it was successful. It effectively reduced disease spread.”

Another said:

“The decision for shutdown—although it limited freedom and caused harm to some groups—it is ultimately in line with society's interests. This type of decision is really ethical.”

Another participant said:

“COVID-19 status is like a wartime status, where difficult decisions must be made to protect people.”

##### Closing schools and change teaching methods

One participant said:

“Although it was difficult, shifting teaching from the usual method to online teaching for millions of students at various stages from primary school to the university, was a huge step but in the correct direction.”

##### Work changes in institutions and companies

Restrictions on gatherings were not limited to the people outside institutions but also inside them, including academic institutions, research centers, and hospitals.

One participant said:

“Our meetings now take place through Microsoft Teams. Teaching lectures as well. This was a positive step because it increases attendance percentages and is highly effective.”

However, this opinion was not shared by all interviewees. Another participant said:

“The quality of performance in these online meetings is not the same as in usual physical meetings, which allows better communication, especially because in the most cases, people don't use video calls but only audio calls.”

##### Prayer, the Umrah, and the Hajj

One of the restrictions that has been widely accepted by the participants is banning communal prayers in mosques and doing them only individually in homes.

A participant said:

“The decision to temporarily stop prayers in mosques was an excellent and necessary decision. It came early, at the correct time, and was effective in preventing disease spread.”

Another said:

“The decision to stop communal prayers is compatible with Islamic law because it prevents harm to others.”

Another participant explained:

“Temporarily banning prayers in mosques was a painful but wise and logical decision.”

This was echoed in the ban on Umrah, a pilgrimage with some similarity to the Hajj, in which many visit Mecca and Medina, the two Muslim holy cities. One participant said:

“Banning Umrah at this time is an ethical and logical decision, and it is in accord with Islamic law.”

One participant said:

“The decision that was made to limit the pilgrimage (Hajj) to very few pilgrims, closer to hundreds than to the usual few millions, was very wise, and it matches Islamic and ethical principles.”

#### Theme 2: Ethical Challenges Regarding Procedures and Actions for Certain Groups

##### Screening of certain groups

The government introduced some restrictions for certain groups that are either exposed to more danger than others or that may cause disease spread. These restrictions included mandatory screening, isolation, and restricted transportation and visits.

One participant said:

“In some crowded collections of people where it is impossible to socially distance and where the health circumstances are not appropriate, screening of these groups specifically is very important and beneficial to the whole community in general.”

Another said:

“Screening is important in some places, such as airports; however, surely we will not stop people in the streets for screening, this is not acceptable.”

A participant said:

“Doing compulsory screening is a serious and painful issue, and we cannot do this in usual cases and forever. We can only do it when there is a strong justification for it.”

One of the participants explained the compulsory screening of specific groups who were poor and living in a lousy environment:

“Doing free of charge screening of these vulnerable persons will help in offering them better protection and more justice.”

##### Isolation

Isolation was applied for 14 days for two groups, namely, those returning from outside the country and those who have tested positive of COVID-19. The majority of interviewees agree on the isolation made by authorities.

One participant said:

“The government has done everything to bring home all nationals who are living abroad and offer them a good isolation environment, including hotels, and good and healthy places to reside.”

Patient isolation is ethical obligation, as expressed by research participants. A researcher said:

“Quarantine limits freedom and social interactions and cause disturbance to social life. We must accept these social effects for good reason.”

Harm prevention is a professional and ethical duty of healthcare providers.

One respondent said:

“If we know that someone is hiding an infection, we cannot simply ignore that. Action should be taken, starting with giving advice to this person and ending with reporting to the authorities.”

##### Protecting volunteers and healthcare providers

The participation of healthcare providers in fighting against COVID-19 is considered to constitute a noble act because they are putting themselves at risk to help protect people and the community.

A participant said:

“Being close to COVID-19 patients—even only to do tests—increases the possible harm to healthcare providers. We should be grateful for and appreciate this kind of sacrifice.”

Protection should be offered for these groups. One participant said:

“Healthcare providers should have sufficient protection because if they get infected, they will put those people around them at risk as well.”

However, no pressure should be placed to force people to do any work that may put them in danger.

A participant said:

“Healthcare provider should not be abused by asking them to do some relevant or risky tasks.”

#### Theme 3: Ethical Challenges of Detecting COVID-19 and Confidentiality Issues

##### Software programs

Some Software programs have been used to help control COVID-19 spread. General tendencies regarding the permissions enjoyed by such programs was expressed by the participants, however, they thought that more conditions should be taken into account. A participant said:

“Software tracking healthy and infected people has benefits; however, to minimize harm, we should review methods of collecting and storing, manipulating, and utilizing information by any third party.”

Another participant said:

“We should know enough and have enough information about any program of this type and its dimensions.”

A participant said:

“The use of this type of software by governments and official authorities can be justified, but never by private parties.”

##### Exposing infected people names

Our participants indicated that revealing the names of people who are infected or in touch with infected people may have some benefit.

A participant said:

“Revealing names is in fact helpful for protecting people.”

However, according to some, revealing names will affect confidentiality and privacy.

One participant warned about the consequences of breaching confidentiality and revealing names:

“Knowing that a certain person is infected or potentially infected may lead to stigma against him.”

A participant said:

“Fear of stigma may prevent others from declaring that they have COVID-19.”

However, another participant expressed a different point of view:

“Stigma about COVID-19 is not strong as it is in other diseases, especially in genetic conditions. On the other hand, stigma can lead to bad results.”

To avoid any negative series of events, some measures should be taken to keep from revealing any names, according to one participant:

“Any revealing of names should only be done by institutions to protect their employees.”

#### Theme 4: Ethical Challenges in COVID-19 Research

##### Quantity of research and publication

It was noted that there has been great interest in research and publishing on COVID-19. For around half of our participants, this raised the question whether we really need all of it. One expressed this concern:

“We have at KAIMRC around 140 research proposals that use different methods and have different objectives. That seems good, but I doubt we need this number of studies.”

Another participant:

“We need more information to develop a better understanding of the virus and the disease, however.”

Another researcher did not see any problem in doing more research. He explained:

“Unlike MERS, which had few patients, the number of COVID-19 patients is large, and therefore they will not be exhausted by the medical experiments conducted on them, as happened to MERS patients.”

##### Uncertainty

The largest challenge in dealing with COVID-19 is that we do not know what information to trust, and every day there is new information coming in that should be judged, discussed, and evaluated separately, although this information may not always be correct, and may even be contradictory.

One participant said:

“Although there is a huge amount of information, articles, lectures, and interviews, we have little concretely grounded knowledge.”

##### Resource distribution

Conducting excessive research on COVID-19 may have a negative effect on resource distribution as expressed by many of the interviewees, which was expressed by one of the participants:

“I do appreciate that we need many clinical trials in order to find medications and develop vaccines; however, this will come at the expense of other research about other diseases. We need to have balance.”

Another participant had a different opinion:

“Here, we have enough resources to conduct all needed research. For example, cancer research did not stop although it costs a lot.”

Another participant considered that the change in resource distribution was acceptable, and he said:

“This is temporary, and everything will come back again after COVID-19 outbreak is finished.”

##### Not following correct experimental path

Hurrying to publish papers may be risky and distort the relationship of mature and misleading information. According to nearly half of interviewees, the desire for rapid results may lead to a push to avoid following accepted steps in clinical trials. One of the participants expressed:

“Clinical trials seeking to find vaccines face the problem that they aren't follow the established stages of vaccine development, including animal experiments.”

The same participant also said:

“The hope of finding effective and safe treatment faces the same problem, namely, passing stages before their time to get results in as soon as possible. This may have negative effects on the efficacy and safety of these new drugs.”

## Discussions

The participants in this study expressed ethical challenges related to fighting COVID-19 especially those regarding preventing public gatherings, social or religious events, and questions. In particular, the challenges discussed related to limiting freedom, economic harms, social impact, ethical challenges related to forced screening and isolation, software that can affect freedom and decision making, and confidentiality, privacy, and the possibility of stigma and discrimination. Moreover, the participants mentioned the ethical challenges that accompany research and studies and how for this type research is beneficial to patients and communities, as well as how resources can be distributed for this kind of research, most importantly not following the correct scientific steps for conducting clinical trials.

In this discussion, we cover different dimensions of these challenges and their effects in the light of ethical standards and principles.

### Facing COVID-19 Is an Ethical Duty

The state has an ethical duty because it is responsible for preventing gatherings and the transmission of disease among its population. Additionally, the citizens have an ethical duty not to participate, whether intentionally or unintentionally, in spreading this disease.

While COVID-19 is still being spread, the community, governments, and individuals must take action to reduce the spread of disease, including but not limited to preventing gatherings, decreasing economic activities, controlling teaching in schools and universities, and taking some other measures. This all is in pursuit of a high and important purpose, namely, to protect people during disasters. This has resulted in a Royal Decree in Saudi Arabia for this purpose, which later played a role in containing the disease the country (22, 23), which falls under the category of due diligence, which have been stated in the general international law (24) and the International Human rights law (25). This matches other aspects of law as well, such as Article 11 of the European Social Charter (26) and Article 12-1 of the International Covenant of Economic, Social and Cultural Rights (ICESCR) (27), among others.

The prevention of gathering also matches the Islamic law that Saudi Law is built on, which mentions the duty to protect persons and prevent harms. An example commonly used in support of this point is early Islamic states, where rulers forced people to isolate to prevent infectious disease (28).

On the other hand, the ethical duties that push people to accept social distancing has a different foundation, most importantly the feeling of responsibility, protecting oneself, avoiding disease, and self-interest, beyond altruism, with little concern for freedom, controlling the population for public interest and to protect others.

This ethical duty and necessity manifest as the need to prevent harm, produce benefits, respecting confidentiality and privacy, increasing justice, freedom, and responsibility, preserving and not wasting resources, protecting the vulnerable, and finally research integrity.

#### Ethical Duties in the Light of Bioethical Principles

The ethical that must be faced in COVID-19, can be summarized as the offer of a maximal amount of protection to society and the community and providing the highest standard of healthcare to patients, following international bioethical principles, which are similar to and largely match the ethical values of Saudi society.

The idea of doing no harm can is the clearest principle to follow for any step in controlling COVID-19, which would include banning gatherings and imposing social distance, and changing teaching to online teaching to offer protection to students and their families. Moreover, temporarily halting prayers in mosques that once hosted them five times per day may be its clearest manifestation, and this can be considered as among the strongest measures, which reflects the government intention to do what is necessary to control Covid-19. This decision received support from the religious authorities and Fatwa bodies (29).

Similarly, stopping the Umrah and reducing the Hajj to a very limited numbers of pilgrims, although it was a major drop in expected income to the state, follow suit. These decisions in the face of economic loss represent the government's desire to take all necessary steps.

Moreover, screening, isolation, and reporting also come under the principle of do no harm, and all were accepted by all of our research participants. These measures are similar to others taken by other countries around the world and are compatible with the recommendations of similar organizations and authorities (30), with the results of other authors (31).

The measure of preventing gatherings is supported by the do no harm principle and also by the beneficence principles, an important item for bioethics. While social distancing policies did contribute to preventing the spread of disease, they also offered protection to people, a direct personal benefit; likewise, institutions that enabled online communication among their employees contributed effectively to protecting them and consequently protecting their interests as a clear benefit.

Moreover, praying at home instead of at mosques goes beyond preventing the transmission of disease to others but also preventing them from being infected. Scanning also goes beyond protecting others, bringing direct benefit to the person who is screened, who can know what steps are necessary to take in the event of infection.

Due to the serious effects of COVID-19, which are increasing in vulnerable groups, such as elderly people, offering protection to them and preventing disease transmission among them should be considered an absolute ethical duty.

However, although social distance in all of its forms, screening, and isolation are important, they nevertheless contradict the autonomy principle, especially the measures are obligatory and compulsory. However, these imperatives should remain at an ethically acceptable level and be included under the headings of both individual responsibility and social solidarity (28).

One of the most important considerations, with comes with the risk of ethical violation, is that of privacy and confidentiality (32). In particular, this should caution against revealing the names of COVID-19 patients, which implies the need for a mechanism regarding revealing names and the importance of this measure (33).

Privacy and confidentiality are also at risk due to the use of software programs for detecting patients on their mobiles, which may be an important method for controlling the spread of the coronavirus (34), such that rapid tracing and discovery of newly infected cases can decrease the spread (35).

The risk will increase as violations occur and stigma or discrimination develops, which can involve risk to the lives of healthcare providers, patients, and survivors (36).

Participant concerns regarding stigma have been noted by other studies (37–39). Fear of stigma and discrimination can result in people hiding their symptoms of their infections, avoiding treatment, and avoiding testing until their condition deteriorates (40), which will have a negative impact on the preventing of disease spread (41).

While performing compulsory screening for all may not be ethically acceptable, doing it for some vulnerable groups may offer them protection and achieve a certain degree of justice (42).

While the participants have many concerns regarding conducting research on COVID-19, social distancing did not negatively affect the number of medical studies. This relates to some questions that faced researchers in their research, such as ethical, legal, and management procedures needed to use information and confidential data (43).

Even though there is a critical need to do research in studies of COVID-19, accelerating them will have negative impact on medical research integrity, as was mentioned by our participants in this study and by mentioned by other authors (44, 45).

The solution comes from finding a balance between the benefits of doing research on the one hand and the risk of offering fast and immature knowledge, which can lead to incorrect clinical decisions, on the other (44).

Moreover, rapid medical research on COVID-19 especially clinical trials may in fact bring a higher degree of risk to participants, which contradicts the ethical principle to do no harm (46).

Doing unnecessary and rapid research can have a negative impact on other resources, especially when those resources are limited, rather than using them in study of other diseases, especially when these diseases have a significant impact.

These issues and ethical challenges in doing research on COVID-19 require the development of mechanisms to review research projects with special committees created for this purpose.

## Conclusion

The participants in this study presented a range of opinions concerning the ethical aspects of the medical response to COVID-19, especially about preventing gatherings, doing screenings, and doing isolation, along with conducting research to develop new treatments or vaccines. The participants recalled the importance of preventing harm, beneficence, protecting vulnerable groups, maintaining confidentiality and privacy, and preventing stigma and discrimination. They also mentioned the important of maintaining research integrity to improve protection and avoid mistakes. However, some other issues remain neglected in our study, such as questions regarding intensive care, which requires further research.

## Data Availability Statement

The raw data supporting the conclusions of this article will be made available by the authors, without undue reservation.

## Ethics Statement

The studies involving human participants were reviewed and approved by The IRB at the National Guard Health Affairs. The patients/participants provided their written informed consent to participate in this study.

## Author Contributions

All authors conceived and designed the study and contributed equally to the writing of this article. All authors contributed to manuscript revision and approved the final version of the manuscript.

## Conflict of Interest

The authors declare that the research was conducted in the absence of any commercial or financial relationships that could be construed as a potential conflict of interest.
